# The Germ Theory

**Published:** 1889-03

**Authors:** 


### Editorial.

### THE GERM THEORY.

We point with pleasure to the photogavure plate in this number of the Journal as an evidence of the marked advance that is being made in our facilities for demonstrating histological conditions. So long as we had to depend upon drawings to represent the different expressions of tissues in health and disease, especially the latter, just so long did there exist the possibility of misinterpretation of the phenomena observed. The matter of personal bias and preconceived ideas of certain conditions were always to be guarded against; but now that we are able, by photomicrography and photogravure work, to reproduce the exact appearances to be found in tissues, either normal or diseased, the whole problem brings itself down to an interpretation of the actual conditions as represented in the negative or plate, by the mass of observers, and not by the individual, as heretofore. It seems to us that the present marks a step in advance, in that the profession at large is introduced into the mysteries of the laboratory through the eye of the camera, and is asked to form conclusions for itself, and not to depend upon the deductions of the single individual, even though he may be ever so painstaking and conscientious.

The evidences are before you, and you are at perfect liberty to draw your own conclusions. It has always been an enigma to us to understand why it was that the profession at large, both medical and dental, should have been so averse to accepting the germ theory of disease when it explained the hitherto unexplainable in many conditions pertaining to retrograde processes, we can only find an answer in a conservatism which has been born of excepting previous theories only to have to give them up in time. Conservatism is a good thing. It keeps us from running away after strange Gods, but conservatism never gave a new idea to the world. To make a real discovery or advance a new doctrine requires a radical.

The opposition to the germ theory grew out of a lack of knowledge of the basal principles that underlay the investigations being made, and the extremely difficult character of the work which prevented many from entering into the field as original investigators. Not wishing to be quiet on the subject, they found it easier to offer obstacles to advance than keep pace with the rapid strides made by those engaged in the work. Had more attention been given to the rules laid down by Koch, regarding the evidence necessary to demonstrate the direct etiological character of any given microbe with a disease, there would have been less opposition from the first. Koch held that it was not only necessary to find a micro-organism as a constant accompaniment to a certain given disease, but that the finder must isolate, cultivate, innoculate and reproduce the disease. Nearly all the work that has been done in his famous laboratory has been done upon that basis; and now the French come in with a line of discoveries relating to the alkaloids produced by certain forms of germ life. The result of their discoveries adds an additional proof to the direct connection of germs to those diseases that have thus been investigated, and puts to rest the long disputed question as to whether the micro-organisms were the cause or effect or diseased conditions.

Breiger has studied fully the ptomaines of peptones, putrescent meats and cheese; Debierre, the infectious diseases; and Bouchard, the products of the uropœtic system. It has been conclusively proven that pathogenic micro-organisms produce, not only toxic alkaloids, but that in a few instances, leucomaines, also, which products act deleteriously upon the system. We must now draw a distinction between *infection* and *toxæmia*. Infection means the penetration into the system of some living principle which has the power of self-multiplication, while toxæmia refers to the condition produced by the product of these living organisms. Ptomaines are the waste products of life and are not equally poisonous to all life, and while the waste product of the germs that produce decay is not, in the strict sense of the word, a ptomaine, because not being toxic in character, yet it is decidedly deleterious in its action on tooth substance. Dr. Miller, in determining the character of the agent, lactic acid, produced by the germs causing decay, was in the front rank in the line of chemical investigations that have since led to such satisfactory results. To conclude, any one to establish his claim as the discoverer of the germ that produces a certain disease, must first prove that the germ is the constant accompaniment of the diseased condition, must cultivate, isolate, innoculate and reproduce the disease and then determine the character of the toxic element that produces the pathological condition. With such precise and exacting conditions can you not accept the result of the labors in proof of the germ theory. Dr. Miller has done all this in his studies on decay, and we have the pleasure of presenting to you ocular demonstration of his researches in the photogravure plate to be found in this number.

